# Exploring Staff Attitudes Towards Unspecified Kidney Donors in the United Kingdom: Results From the BOUnD Study

**DOI:** 10.3389/ti.2023.11258

**Published:** 2023-06-08

**Authors:** Mira Zuchowski, Nizam Mamode, Heather Draper, Peter Gogalniceanu, Sam Norton, Joseph Chilcot, Timothy Auburn, Alexis Clarke, Lynsey Williams, Lisa Burnapp, Paul McCrone, Hannah Maple

**Affiliations:** ^1^ Department of Psychology, Institute of Psychiatry, Psychology and Neuroscience, King’s College London, London, United Kingdom; ^2^ Department of Transplantation, Guy’s and St Thomas’ NHS Foundation Trust, King’s College London, London, United Kingdom; ^3^ Health Sciences, Warwick Medical School, University of Warwick, Coventry, United Kingdom; ^4^ School of Psychology, University of Plymouth, Plymouth, United Kingdom; ^5^ Directorate of Organ and Tissue Donation and Transplantation, NHS Blood and Transplant, Watford, United Kingdom; ^6^ Institute for Lifecourse Development, University of Greenwich, London, United Kingdom

**Keywords:** living kidney donation, unspecified donation, unspecified kidney donor, non-directed, altruistic donation

## Abstract

Unspecified kidney donation (UKD) has made substantial contributions to the UK living donor programme. Nevertheless, some transplant professionals are uncomfortable with these individuals undergoing surgery. This study aimed to qualitatively explore the attitudes of UK healthcare professionals towards UKD. An opportunistic sample was recruited through the Barriers and Outcomes in Unspecified Donation (BOUnD) study covering six UK transplant centres: three high volume and three low volume centres. Interview transcripts were analysed using inductive thematic analysis. The study provided comprehensive coverage of the UK transplant community, involving 59 transplant professionals. We identified five themes: staff’s conception of the ethics of UKD; presence of the known recipient in the donor-recipient dyad; need for better management of patient expectations; managing visceral reactions about the “typical” unspecified kidney donor; complex attitudes toward a promising new practice. This is the first in-depth qualitative study of attitudes of transplant professionals towards UKD. The data uncovered findings with strong clinical implications for the UKD programme, including the need for a uniform approach towards younger candidates that is adhered to by all transplant centres, the need to equally extend the rigorous assessment to both specified and unspecified donors, and a new approach to managing donor expectations.

## Graphical Abstract

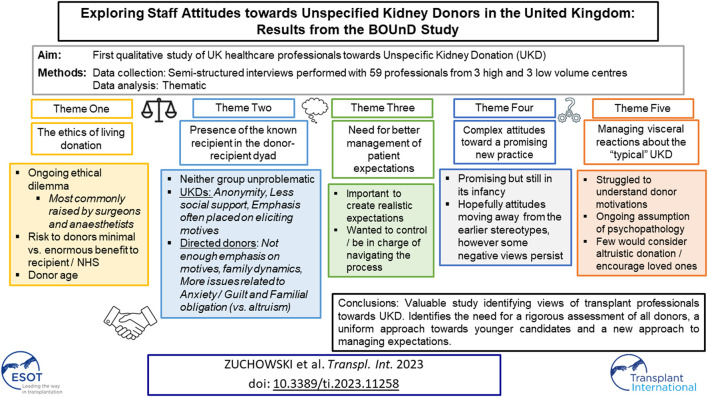

## Introduction

Living kidney donation (LKD) is the gold standard treatment for End Stage Kidney Disease [1]. LKD benefits the recipient, who experiences an improved quality and duration of life, and reduces pressure on waiting lists. Kidney transplantation in general reduces the economic burden of renal replacement therapies, thereby allowing healthcare resources to be redistributed more efficiently [2]. In the United Kingdom (UK) there are two pathways to LKD: specified kidney donation (SKD) to a recipient known to the donor, and unspecified kidney donation (UKD) from an unknown donor to an anonymous recipient [3]. Unspecified kidney donation accounts for around 7%–9% of the UK living kidney donor programme and has made a significant contribution, both directly and as part of the UK Living Kidney Sharing Scheme (UKLKSS). Unspecified Kidney Donors (UKDrs) are used within the UKLKSS to trigger a chain of transplants (called “altruistic donor chains”) between 2 or more incompatible donor–recipient pairs. The remaining organ from the donor at the end of the chain is then allocated to a recipient on the national transplant list [1].

Despite this, some transplant professionals feel uncomfortable caring for these individuals, mainly due to concerns that wishing to donate is a manifestation of an underlying psychopathology [4, 5]. Consequently, a mandatory and rigorous psychological assessment is undertaken in all UKDrs [6]. Such an assessment is optional for specified kidney donors (SKDrs) and is at the discretion of the individual case or transplant centre. The programme also remains controversial because there is a general lack of data on outcomes and other aspects due to its relative novelty [1, 7, 8]. Concerns have been raised about whether the UKD programme in its current form represents an optimal use of NHS resources. This concern is based on anecdotal reports that UKDrs receive more meticulous and lengthy screening than other Living Kidney Donors, thus creating additional healthcare costs. UKD raises a number of ethical concerns for medical professionals, primarily the dilemmas around subjecting a healthy individual with no connection to the recipient to a serious operation. For these reasons, some healthcare professionals may have concerns that could influence the messages that they convey to potential donors.

A qualitative study exploring the experiences of UKDrs suggested that some participants were distressed and confused by discouragement from healthcare professionals, and the study highlighted the desirability for consistent messaging from staff members [9]. Participants also reported feeling distressed by the rigorous mental health assessment, believing that their motivations and overall sanity were being judged [9]. One study has explored transplant physicians’ views on the nature of altruism in UKDrs and questioned whether it existed [10]. We therefore wished to explore the attitudes of healthcare professionals in the UK towards unspecified kidney donation, as well as to investigate whether there were barriers to donation.

To our knowledge, this is the first study to provide an in-depth exploration of the attitudes of UK transplant professionals towards UKDrs, and forms part of the Barriers and Outcomes in Unspecified Donation (BOUnD) study, which is exploring the barriers to UKDrs in the United Kingdom [8]. A qualitative study was performed to determine potential issues that are not necessarily apparent in questionnaire-based research. The aim of this study was to investigate the broader views and experiences of the UK professional transplant community towards UKD, and explore to differences between centres, and different members of the multidisciplinary team.

## Patients and Methods

### Participants and Setting

The participants in this study were recruited as part of the BOUnD study [8]. Funded by the National Institute for Health Research (NIHR), staff and patients were recruited from all 23 UK transplant centres. To explore the attitudes of UK transplant professionals in more depth, a sub-study recruited staff from six UK transplant centres: three high volume centres and three low volume centres. Centres were defined as high or low volume based on UKD numbers at these centres in 2016/17 [11]. Analysis of national data demonstrated that approximately 50% of UKDrs donated at five of the 23 transplant centres. Centres were grouped according to numbers of UKDrs and those with the highest and lowest total numbers were approached. Using opportunistic sampling, representatives of staff groups involved in the UKD programme were recruited, including but not limited to, transplant co-ordinators, nursing staff, nephrologists, clinical psychologists, and surgeons.

### Interview Topic Guide

Semi-structured interviews were conducted according to a topic guide. This was developed based existing literature on the topic and staff focus grouped performed as part of BOUnD. The interview topic guide covered:

1) Terminological preferences for UKDrs
2) Staff perceptions of UKDrs and thoughts on their specific motivations
3) Staff perceptions of their own work with UKDrs
4) Perceptions of the transplant professionals working with UKDrs and how treatment differed to SKDrs
5) Opportunity to reflect and provide suggestions for developing the programme.

Telephone and in-person interviews were conducted by two researchers (authors 8 and 9).

### Qualitative Analysis

All interviews were audio-recorded and transcribed verbatim. The interviews were anonymised, and full transcripts were circulated to members of the study team (authors 1, 7, 8, 9, 12). The data was analysed using NVivo 11 Plus software.

An inductive thematic analysis of the data was conducted. This methodology was chosen because it is data-driven in nature and not linked to any pre-existing theoretical model [12]. It is considered suitable when investigating a diverse data set that is expected to reflect a broad range of attitudes towards the research questions [12]. The analysis involved multiple consecutive readings of the transcripts in order to become familiar with the data and to identify and code themes and categories and highlight relevant patterns across the data set [13, 14]. The next step was to analyse the codes and consider how these could be grouped thematically to encompass a range of ideas around a common topic [15]. This grouping of codes into themes and sub-themes was the product of repeated discussion between the coder (MZ) and the research team (HM, SN, JC). The analysis conformed to the COREQ (Consolidated criteria for reporting qualitative research) checklist [16]. In order to ensure reliability and eliminate preconceptions about the data set, the analysis was conducted blind.

## Results

59 interviews were conducted between April and November 2016. Thirty were from high volume centres and 29 from low volume centres. The average interview length was 32 min (Range: 10–76 min; SD = 15.33).

### Participant Characteristics

**FIGURE 1:**
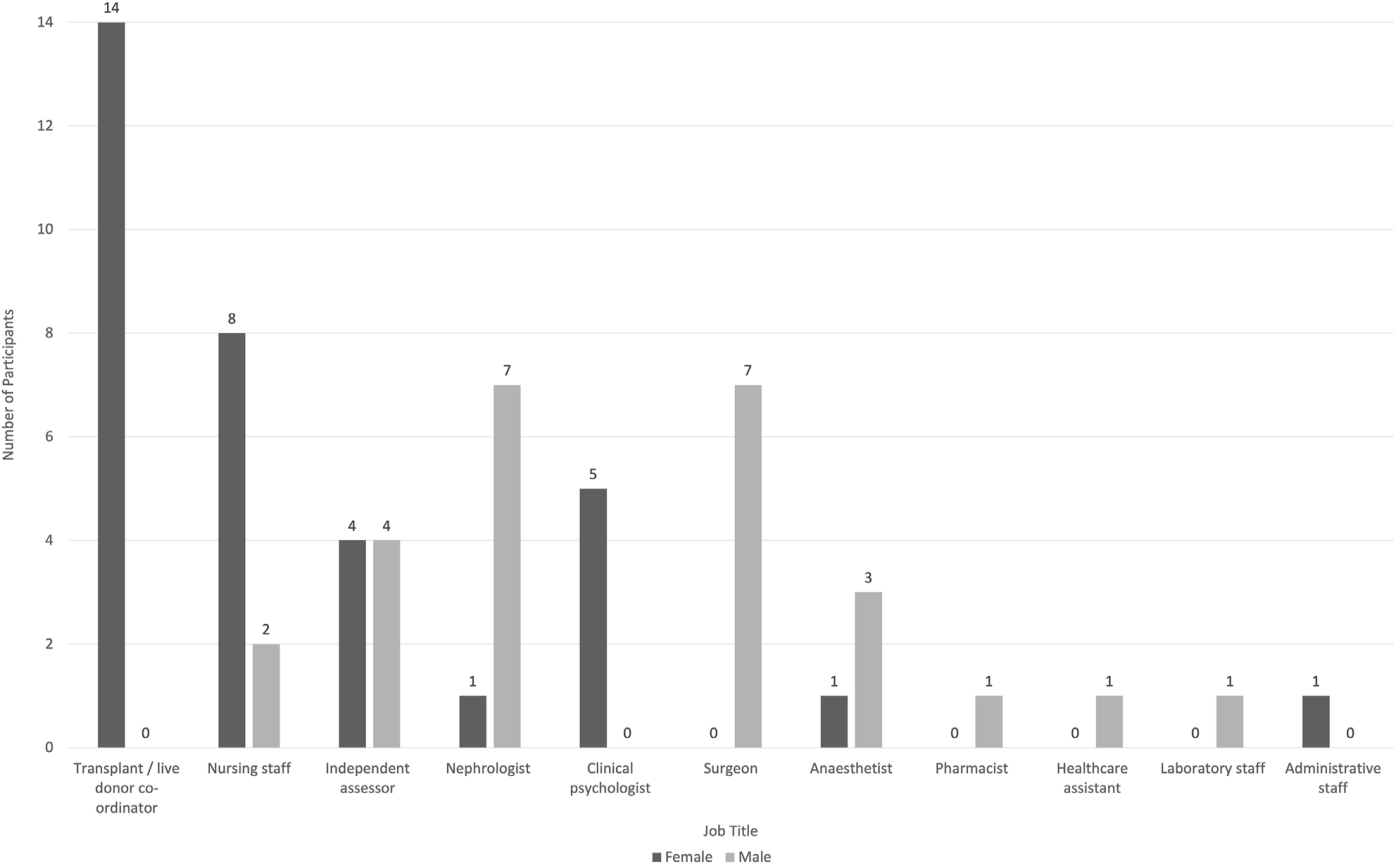
Participant characteristics.

The study provided broad coverage of the UK transplant community. The majority of participants were women (57%), and the most frequent professional roles were transplant coordinators (20%), and nursing staff (17%) (Figure 1).

### Staff Attitudes Towards UKD

**FIGURE 2:**
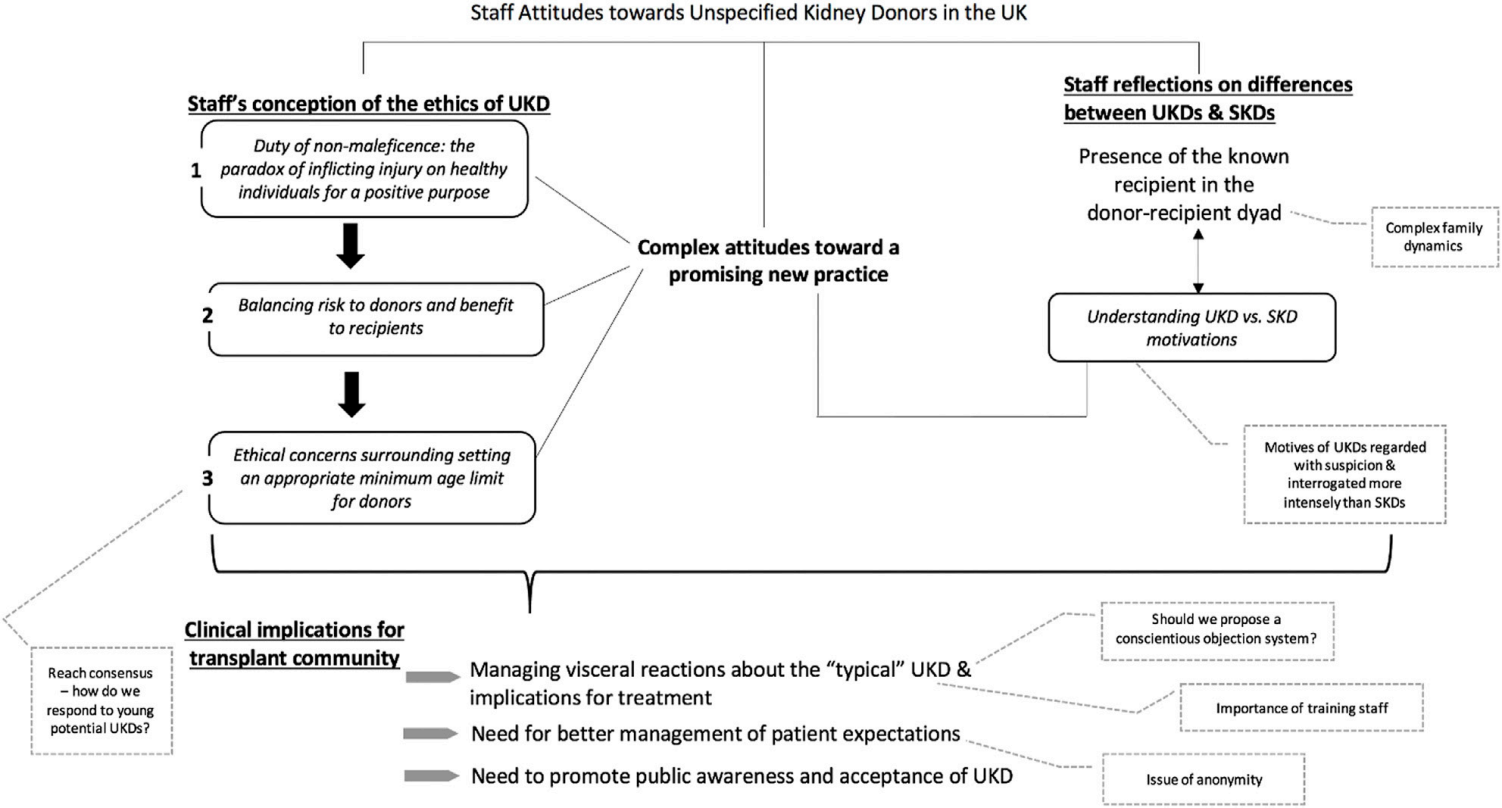
Thematic diagram.

Five major themes emerged from the data [1]: staff’s conception of the ethics of UKD [2]; presence of the known recipient in the donor-recipient dyad [3]; need for better management of patient expectations [1]; managing visceral reactions about the “typical” UKD and implications for treatment and [4] complex attitudes toward a promising new practice. Each theme and corresponding sub-theme(s) are discussed in detail below (Figure 2). Table 1 provides supporting quotations.

**TABLE 1:** Themes with corresponding subthemes and quotations (H—denotes high volume transplant centre, L—denotes low volume transplant centres).

Theme/Subtheme	Quotes
1. Staff’s conception of the ethics of UKD	“As long [as they do that and] the process is informed, I don’t think it raises any additional ethical issues over and above that.”—H
	“I am a big believer in if you want to do something and you have got the capacity to consent to it, that you should be allowed to do it, and to that point I even find my role a little bit difficult because I think … who am I to suggest that this person might not be able to do something they want to do?”—L
	“I don’t see it any different from somebody donating blood in the sense of … once you have stepped over that point, then I can’t see what the difference would be… That would be my simplistic answer.”—L
	“It’s ethical as long as there’s been a full psychological, maybe psychiatric assessment of that person and I think for me that is the biggest, because there are some very lovely people out there that just want to benefit mankind and so if they go in there and they have no psychological or psychiatric drivers, then I think it’s a very magnanimous thing to do”—H
	“You can live quite happily with one kidney as long as everything is all right. I haven’t got any ethical problems with it at all. If someone wants to do that, why not?”—H
1a. Duty of non-maleficence: the paradox of inflicting injury on healthy individuals for a positive purpose	“I think it’s always the stress, like you say, of operating … more for the surgeons, of operating on someone who is completely well, you know, and in some ways it’s easier just to deal with an emergency when someone is bleeding.”—H
	“I am not sure whether we should actually ethically be doing this, because doctors and I guess any healthcare professional is supposed to do no harm and these people are specifically … we are allowing them to put themselves in harm’s way and not even, you know, to benefit themselves or their family”—H
	“It comes down to ‘doing no harm,’ and my understanding is that the Hippocratic Oath doesn’t have that as part of it; it’s something that came much later in medicine … I am a nephrologist, but I have seen a lot of harm to a lot of patients over the years—unintentional, drugs being prescribed, wrong doses given, infections not being dealt with properly, symptoms not being listened to … so the world is clearly not perfect. And I think most patients I look at, say people who come forward, understand that something for nothing doesn’t occur in the real world. They realise there is risk, most of the people I have come across are willing to take much more risks than others and I don’t think it’s a misunderstanding of risk, I think it’s actually doctors working professionally acting on behalf of the people who tend to be more risk-averse than those individuals I think.”—H
	“Although medical ethics to do no harm, you know, is a bit old hat I think now, 50 years on, I am sure from a recipient’s point of view, the fact that altruistic donation is now permissible within the law, makes an enormous difference for them because it’s just, you know, an extra chance, one opportunity.”—H
1b. Balancing risk to donors and benefit to recipient	“I don’t have any problem with the ethics because you are doing something which is a small risk to do quite a large good … I think it is a question of looking at how much good you are doing in somebody who has got a normal personality and is not mentally ill.”—L
	“we have a 7,000 patients waiting, so if we can get, you know, some altruistic donors, that will have a great impact on our waiting list.”—H
	“I think that the consensus view is that it is appropriate for an individual to perform an act like this for the grander good, principally for the good of another individual. As I say I think the overarching ethic of that is quite appropriate and I can work with that.”—L
	“we are reducing the number of people who are on the waiting list. If we get more altruistic donors to donate their kidneys … hopefully people will be getting their organs quicker and we will reduce the number of people on dialysis”—H
	“what we have forgotten is actually that not only are people going away with new kidneys and a brand new life, but also you are freeing up dialysis for other people so it’s almost a double whammy. You have got people starting a brand new life without the shackles of dialysis, be that at home or in a unit, they go off to start this new life and then you have also got these free areas for people to come and start dialysing”—H
	“you see people you know coming off dialysis and getting a fantastic gift so that’s very beneficial.”—L
	“it’s more rewarding because of course you are maximising transplant opportunities for other people in the pool by the fact that maybe two or three people through the paired scheme, they get transplanted”—H
1c. Ethical concerns surrounding minimum age limit for donors	“I think my concern is with youngsters they can be 18 to 30 and I know it’s a broad age group and it’s a broad age range but they’re still developing and maturing and it’s ensuring that they understand what they are doing, it’s not just a good idea or a nice thing to do. Have they thought through implications.”—L
	“For me, the biggest dilemma I had, we were approached by an 18 year old which felt uncomfortable. It was a female so of course then there’s the additional of … of course she didn’t want a family, the minute you are 18 you don’t think further than tomorrow, so she hadn’t thought about babies and the implications of having a single kidney around pregnancy and things like that … She had got obviously a long road ahead of her with potentially just a single kidney. So I was really uncomfortable with it, although the protocols said it was fine to carry on.”—L
	“We all have concerns about the youngsters coming forward. Some of them … have had some sort of mental health issues, organise a psychiatric review and I think because they have all dealt with a lot more than I have, and they have seen a lot more, I think I am gathering my experience so they compare to the others in the team, but there is a lot of concern about the young ones coming through and their motivation.”—L
	“I’m worrying about 10 years time, 20 years time—all the young ladies that come through that haven’t had families, you know, how are you going to…? Are we going to facilitate damage to them? Is it going to be something, for example, when they have a baby they can’t then”—H
	“We have seen people, for example, donors who are very young, a 23 years old coming forward as an altruistic donor, for me I am a bit conservative. What do you know at 23? You are only 23, what do you know about life? … I have got a 27 years old son and I know what a boy of 27 … and coming forward at 23 and ‘You say you want to be an altruistic donor?’ That’s early, I would say ‘Please it can wait’?”—H
	“Quite often other members of the team in particular will say ‘I don’t feel very comfortable about this, they are only 19 or they are only 21’ … but I don’t share that view … young people make decisions, sometimes those decisions may not be wise decisions … you are young you can still get drunk, drive a car, crash it, get pregnant, have a tattoo, all sorts of things which I might not agree with but you’re still legally entitled to do it and I think an altruistic donor is entitled to make a decision even if they are only 18 or 19. I don’t think it’s for me to say ‘Oh you are too young, you don’t know what you are talking about’ so I don’t share the anxiety of the other team members.”—H
2. Presence of the known recipient in the donor-recipient dyad	“There is an issue isn't there, if you have donated a kidney and that kidney doesn’t work for whatever reason, like if a husband donates it to … or a mum to a child, and it didn’t work, the guilt that you would feel for that not working. But if it’s an altruistic you wouldn’t really necessarily know what had happened and how that was going on and whether the control from you”—H
	“The relationship is part of the meaning. It can be that the relationship … well it’s making them feel guilty about … had made them feel forced to do it, but actually what we found out is they don’t want to do it at all … so there’s different processes that happen, no, it’s not straightforward, it’s not … It can be, often less straightforward than the altruistics”—L
	“I think they have got no vested interest really, emotional interest in the recipient’s wellbeing. They have given their kidney and I suppose as soon as they have given their kidney they feel as though they are in the right, their job has ended. You know, it’s not as if they had to take care. They don’t provide a carer role or a supportive role to the recipient so from that point of view they are different I suppose.”—H
	“some of these altruistic donors afterwards, they do expect something back from the recipient, so when they don’t hear something back from that recipient, even just to say ‘Oh OK, we are OK’, they find that very difficult”—H
	“that is a question of you can never say to them ‘I did this for you, so you have got to do this for me later’ because there is a pressure/coercion bit that can happen post-op…. we always assume that it’s going to be offered up front at the beginning but it might be that the donor later says ‘Well I did that for you and you are not being really helpful and fair now,’ you know, ‘mum has left you 60% of the inheritance and I am only getting 20% because the other 20% is going somewhere else, how about upping mine by sharing?’ ‘Well why did I do that?’ ‘Well, look what I did for you’—you can hear it can’t you?”—L
2a. Understanding UKD vs. SKD motivations	“But my personal feelings on altruistic is it doesn’t really exist. I think everyone gets something out of it, I don’t honestly believe they are doing it just for the good of others, and even if they are quiet about it and they’re not standing on a soap box going ‘Look at me, I have donated a kidney,’ they must internally get some sort of validation or some purpose from doing that.”—H
	“I think altruistic by strict definition to me is a truly selfless act and I think there are very few things in life that are truly selfless acts and I don’t think altruistic donation is one of them”—L
	“I have … reservations about the human’s ability to be truly altruistic and whether we are facilitating some form of process by which there’s either cathartic process or some form of other process going on that we are facilitating in the name of altruism”—H
	“I think the ethics are very difficult aren’t they, because again it’s touching on what is true altruism, what really is the endpoint for what people hope to get out of donating a kidney? As I said before we have been stung here within the last year and we got some very bad press from somebody who you can argue therefore isn’t altruistic, so you could say are we using the right word when we call it altruistic donation? If we didn’t use that word then the ethical issues may not be quite as large.”—H
	“The differences, we over-cook the altruistic, we do, we do, particular on the psychological side”—H
	“They do have a psychological assessment … and I think that’s really important to get that right because if they have got an ulterior motive or if they are going into it not necessarily 100% sure of what they are actually doing, then that could potentially lead to problems”—H
	“They don’t automatically get a psychological assessment if they are a live donor pair, whereas an altruistic we always ask for them to be assessed.”—L
	“I think people also think people do it on alternative motives, I don’t think people can quite believe that somebody would just do it altruistically.”—L
	“More the issue around directed donation is an element of … if you think there’s an element of coercion. So occasionally you will see a family group come in and I remember this recently … the brother said ‘I don’t know why I am here, my sister told me to come, I am sorry but I really don’t want to do this’ so that’s more the get-out in the directed side, someone who has come along because they don’t feel they can say no.”—L
3. Need for better management of patient expectations	“Because that relationship isn’t there for the non-directed altruistic donor, it’s absolutely essential that they have a full understanding of the risks so I do spend a lot more time with them talking about risk … Whereas with the directed donor, if it’s a complex paediatric case or someone with a medical problem where the disease might recur, I will give much more tailored information and say ‘Look, this is a really high risk transplant for this recipient, you need to be aware of x, y, z’ and then we will have a discussion around that with the directeds whereas we can’t do that with the undirecteds.”—H
	“The downside I think is managing … I think it’s more about managing the post-op and managing people’s expectations and as I say, I think not having, not seeing … they are often really keen and ask all the time how the recipient is doing, because they are often not in this hospital they don’t get that feedback”—L
	“their expectations will probably be one of the sort of difficult things to manage … it’s so important we give them as much information as we possibly can. It might be worth just asking the sort of individual as part of their process, what did they expect as part of the outcome of this? Would they expect the recipient to be in touch? You know, I think establishing what their expectations would be.”—L
	“Well I think directed donors, they have someone specific in mind so they have got that motivation, they know that person whereas from an altruistic they don’t know that person … I think they have to have those expectations clearly put out at the beginning. They need to be prepared a lot more I think.”—H
	“the challenges are … actually making them accept the risk because they are just like ‘Oh it’s fine. If you are telling me I can do it, I can do it so that’s fine,’ ‘but it still comes with risk’—that’s what you find. Whereas it's not an emotive ‘but it doesn’t matter if something happens to me, I want my loved one fine’—they haven't got that loved-one pull like I keep saying but you … ‘Are you really listening? You know, ultimately you could die under anaesthetic’ ‘Yeah, yeah, yeah that’s fine’ and I just think…. ‘well don’t just say ‘Yeah, yeah it’s fine, are you really listening, are you understanding that point you know, you don’t have to do this?’”—L
4. Managing visceral reactions about the “typical” UKD and implications for treatment	“I am sure they have their own reasons for making that decision, but personally I find that it’s a very difficult decision to understand … I think the only thing is, I still struggle with why anybody would want to do it. I wouldn’t!”—L
	“Because you think if you want to help people you could go and volunteer at a soup kitchen or, you know, once a week instead of drastically being operated on and having an organ removed.”—H
	“I have never heard of anybody being put off by an abrupt doctor or, you know, a rude psychiatrist or something, I have never heard of anything like that.”—H
	“At what point are we facilitating some form of pathological behaviour”—H
	“I think as a group it’s easy to look at them and think they are all strange, with some hints of, you know, mad behaviour.”—H
	“There are some who have pathological traits to them and it’s those that I am trying to ensure don’t give”—L
	“I think you are more likely to get a pathological personality offering it than you would … statistically, than somebody giving it to their spouse for various reasons.”—L
	“they are not very easy to work with because they have unrealistic expectations, they think that OK, I am here to give you an organ and because I am a special donor you have to treat me specially, and we do treat all our donors specially because they’re all special people”—H
	“Some of them have proven to be mentally unstable”—L
	“I do think a lot of people think people are mad and I think that people think why would you do that?”—L
	“I do sometimes wonder if we should even be doing it … when we first started I think, I think maybe some healthcare professionals (myself included) felt it was a dubious decision mainly in that anyone that came forward to do it could be considered slightly mad.”—H
	“I just think we have all, individually, had experiences of altruistic donors being slightly mentally unstable or not predictable or … I would say needy afterwards actually, and I think a lot of them have proven to be attention-seeking, self-publicity seeking and are rather daunted by their lack of attention or lack of emotion given to them afterwards”—L
5. Complex attitudes toward a promising new practice	“I think we … as a transplantation community need to think about where altruistic donors fit in as well. I think there is a conception that these altruistic donors don’t necessarily go to the fittest of recipients because they are altruistic donors and I think there is a danger that we could see them as a second-rate donor compared to directed donors perhaps … I think as a transplant community I don’t think we have quite worked out where altruistic donors fit in, that they could be directed towards patients who weren’t necessarily a last resort really, that somebody may actually get more benefit from these kidneys.”—L
	“I think we are all a bit … I think altruistic donation as a viable source of organs is still actually very much in its infancy, and we do such small numbers, and I think people’s opinions of it will change if we continue to increase in the numbers”—L
	“I think there has been a change actually because I can remember when … the first time I did an altruistic donation, it’s quite a few years ago now and I remember … one of the coordinators saying ‘It’s a bit odd, she has offered, this woman has offered, I don’t know why. Why would anybody offer a kidney? It’s such a big thing, you know’…And then as the years have gone by, I suppose 3 or 4 or 5 years ago now, they have said … now, it’s ‘Oh we have got a altruistic one and there’s been a change, you know.’”—H
	“So whenever there is a new programme people are understandably slightly cautious so it’s partly a temporal issue, it’s a fairly new thing, it’s only been going for what, 6/7 years I guess? That’s partly it, so from an infrastructure and legal perspective”—H
5a. Need to promote public awareness and acceptance of UKD	“Letting people know about it … I think everybody I have spoken to pretty much has heard about it on the radio or TV, or has known somebody who has had a kidney problem so has investigated it. I don’t know how you would know about it otherwise, but I know people I talk to don’t have any idea that it’s something you can do.”—L
	“As with everything, get out there and education. The more people know or see some good results … well the problem is your anonymity with the recipient and things like that, but there’s nothing like good story stuff to make people think that that’s perhaps that’s something they could do. Education, I mean things like the advertising on telly.”—L
	“Well I mean the only thing would be a publicity campaign. I mean I think you could do … if you got it on national telly after Emmerdale or Coronation Street, you know, then … that’s what you need, you need a big publicity programme because actually if you had … I can’t remember the statistics but you could actually solve the waiting list dilemma completely if you had 1,000 altruistic donors a year rather than 100.”—L
	“I think a lot of it is to do with promoting living donations continuously and also the different aspects, the different options we offer in that direct donation obviously and then there’s the paired pool sharing scheme so providing more awareness to the public that way, and I am sure there are other ways as well.”—H
	“we should utilise our … the ones, the individuals that have basically donated, it would be nice to utilise them a little bit more in campaigning. ‘Actually we can do this, these individuals have done it, and they are doing very, very well’ and their stories I think would be more beneficial to the media and the public to see that actually you can do this great deed and still live a normal life as well”—L

#### Theme 1: Staff’s Conception of the Ethics of UKD

Many staff expressed the view that UKD is ethically unproblematic. They had an overriding awareness of, and commitment to, ethical principles and their role within transplantation and living donation, and for the most part felt that UKD fell within those ethical parameters. However, the data remained heterogenous on this topic, resulting in the following sub-themes.

##### Duty of Non-Maleficence: The Paradox of Inflicting Injury on Healthy Individuals for a Positive Purpose

It was apparent that whilst some participants perceived operating on healthy individuals as an ethical problem, others did not. This was most commonly raised by surgeons, although many did not think it was a decisive reason against UKD. This was mainly due to recognition that the benefits of UKD outweighed the harms, providing the donors were fully informed, aware of the risks, and that they had sufficient capacity to consent. Some regarded the concern as outdated. Many doctors tended to express a sense of awareness of the paradoxical nature of their actions, i.e., the dilemmas of a healthy individual undergoing unnecessary surgery, albeit for a greater good. Many healthcare professionals did not think that their ethical reservations influenced potential donors.

##### Balancing Risk to Donors and Benefit to Recipients

Whilst staff members acknowledged the risks of donation, they were commonly weighed against the benefits, which they felt clearly favoured UKD because of the benefit to recipients and other aspects of the healthcare system. They emphasised the overriding benefit of avoiding dialysis and freeing up dialysis facilities for new patients, and to start a new life. Across all centres the prevailing attitude was that as long as people were psychologically and physically fit to be donors, the risks to the donor was minimal in comparison to the benefit to the recipient.

##### Ethical Concerns Surrounding Minimum Age Limits for Donors

Many staff expressed reservations about encouraging UKD amongst young individuals; referring to people in their mid-twenties. Concerns were related to their ability to provide informed consent and that they may not fully grasp how the risks could affect them later in life. Some participants brought up concerns for women specifically, due to potential implications around pregnancy that perhaps may not have been considered by younger women. Some related the decision to their own children. Others felt uncomfortable discriminating on the basis of age, with some centres having a minimum age restriction and others not. Some did not think that age should affect suitability whilst others were very strict with this criterion. Concern was also expressed that younger people might be more susceptible to media messaging and therefore more easily influenced and impulsive in their decision-making process.

#### Theme 2: Presence of the Known Recipient in the Donor-Recipient Dyad

Many participants expressed the view that a major factor influencing their attitudes was the presence of a known vs. unknown recipient. Some staff members said that the donor-recipient relationship in some cases made the donation process more difficult for the staff due to presence of complex family dynamics. They commented that, in some respects, UKD was more straightforward because of the absence of a relationship between donor and recipient. However, there was a notable lack of consensus on this issue. For some staff, UKD presented more difficulties than SKD due to issues such as UKDrs struggling with the requirement for anonymity from the unknown recipient or lack of support network for UKDrs. Overall, however, there was a greater perception that SKD was more emotionally complex due to emotional and physical proximity between the donor and the recipient, and therefore associated with issues such as anxiety, guilt and familial obligation (as opposed to altruism).

##### Understanding UKD vs. SKD Motivations

The role of altruism as a motivator for UKD was questioned by some participants. The emphasis often placed on the mandatory psychological assessment by professionals was considered to be important not only to elicit a UKDrs’ psychological state, but to further clarify their motivation to donate. Some found UKDrs’ motivations to be complex or unclear as, at times, it was difficult to know if candidates were purely selfless or self-interested. Some staff noted that less attention was paid to the motivations of SKDrs, and the potentially complex family dynamics and psychological impact on both the donor and recipient.

#### Theme 3: Need for Better Management of Patient Expectations

Many professionals emphasised the importance of creating realistic expectations for the UKD process: the rigorous psychological assessment, the risks associated with the operation and recovery and the potential emotional consequences post-donation. Anonymity was raised as an issue, especially with regard to the negative emotions that may be experienced should there be no acknowledgment from the recipient and the need to prepare UKDrs for this, as it may present more of an emotional challenge for donors than anticipated. It was also stressed that donors should be informed of these issues from the very beginning of the process. Overall, UKDrs were thought to underestimate surgical risks and wanted to maintain control of the process and be in charge of navigating it.

#### Theme 4: Managing Visceral Reactions About the “Typical” UKD and Implications for Treatment

Many participants admitted that they struggled with understanding why UKDrs come forward. Despite their roles facilitating living donation, some said that they would not themselves consider donating as a UKD or encourage family members to do so. Some participants reported that they did not think that their personal opinions influenced self-withdrawal. UKDrs were referred to by some as being a mentally unstable group.

#### Theme 5: Complex Attitudes Toward a Promising New Practice

UKD was generally regarded as still being in its infancy and that peoples’ attitudes may change once more people donate and transplant professionals have more experience. Some transplant professionals said that there was a need for the transplant community to understand where UKDrs fitted in within living donation. Comments could be reasonably interpreted as suggesting that UKDrs were not as highly valued as SKDrs.

Those working in lower volume centres, who consequentially had less experience, felt that they were unable to make specific generalisations about how they perceive UKDrs as an overall group. Across all centres participants tended to acknowledge that living donor kidneys were the best option for someone on the waiting list (when compared to deceased donor kidneys), and that UKD was a promising and growing avenue for live donor transplantation. There was some impression that attitudes were moving away from the earlier stereotypes of UKDrs being driven by pathological motives, although these views persisted and were still quite commonly held.

##### Need to Promote Public Awareness and Acceptance of UKD

Almost all the staff members who stated that they were broadly in favour of the UKD programme suggested the need to find better ways to promote it amongst the public.

They expressed the view that this would both increase numbers and ensure that future potential donors fully understood the process before offering to donate, therefore reducing dropout rate and conserving resources. Many staff members referred to the effectiveness of utilising past donors in public awareness campaigns, as well as publicising the experience of both donors and recipients.

## Discussion

This qualitative interview study explores the views and experiences of UKD participants drawn from the professional transplant community in the United Kingdom. It provides an in-depth analysis of 59 interviews, currently representing the largest qualitative study investigating transplant professionals’ attitudes toward UKD. The main findings are that many participants expressed reservations about proceeding with younger potential donors and favoured specifying a minimum age limit that is higher than the current legal minimum (18 years old). Additionally, many staff expressed concerns about the psychological stability of UKDrs and found their motivations to be complex or unclear. Many staff raised the need to manage UKD expectations particularly around communication with recipients. Finally, the results demonstrate that some healthcare professionals did not think that their personal opinions influenced voluntary self-withdrawal by UKD candidates.

Over the past decade, transplant professionals have criticised the ethics surrounding LKD [17–19], primarily due to the obligation of the principle of non-maleficence. The present study probed the ethical concerns that medical staff must balance when considering all aspects of LKD. Most participants, whilst they still may not be completely comfortable with UKD, recognised that the potential benefits outweighed potential harms, and acknowledged that UKDrs undergo a rigorous assessment process, including a thorough psychological assessment. It was noted that amongst the various roles covered in this study, it was predominantly surgeons who raised the ethical concern of operating on healthy individuals most frequently. We speculate that this is because they are ultimately responsible for the physical act and are answerable should complications occur.

Another ethical consideration was related to donor age; specifically the concern that younger donors may not fully understand the longer-term implications of their decision to donate. Previous research has explored whether minors and young adults should be legally permitted to qualify as donor candidates [20–23]. Using qualitative methodology, Thys et al. (2019) found three reasons for a cautionary view of living donation by minors and young adults, which were all echoed in the present study: concern about the long-term medical and psychosocial risks of donating a kidney at a young age, younger donors’ capacity to make informed decisions, perhaps related to their developmental stage and the possibility of younger individuals’ greater susceptibility to familial pressure. Similarly, the present study highlighted the ethical dilemmas surrounding age of donation in UKDrs specifically. One emergent concern, specifically for young women, was the possibility of complications related to pregnancy [24, 25]. Our findings suggested there is an inconsistency between transplant centres in the approach taken to younger candidates. As things stand, younger potential donors who are turned down on the grounds of their age by one centre could present to another centre for a different outcome. A national consensus on a minimum age limit or alternatively transparent regional variation would be preferable. Transplant units should publicly clarify what their local policy is both for staff members and potential donors.

One critical issue that emerged from the data was the complexity around the role of altruism within UKD, and why staff placed an overwhelming emphasis on it when discussing UKDrs’ motivations. In the UK, all LKD candidates must undergo evaluation by an Independent Assessor on behalf of the Human Tissue Authority (HTA) in order for the donation to be legally approved. The HTA refer to “altruism” as a means of distinguishing between types of donor, rather than it being a prerequisite for UKD. Our findings indicate that staff attach more weight to the concept of “altruism” than the minimum standard applied by the HTA. In fact, almost all staff reported that they referred to UKDrs as “altruistic donors” even though some donors prefer the term “unspecified” [26]. We question how important it is that donors are motivated by “pure altruism,” as opposed to what might be seen as less selfless reasons. For example, staff members cited a range of motivations they had seen, including war veterans giving back if they have taken a life in the past, individuals atoning for bad behaviour, relationship with renal failure patients, or people seeking religious “credits.” Whilst none of these can be characterised as strictly altruistic we argue that likewise they cannot be put in the same category as receiving material or financial benefit. Previous discussions in the transplant literature demonstrate inconsistency in the way the principle of altruism is applied to living donation [27, 28]. Saunders (2012), for example, argued that while rejecting certain questionable motivations, it is short-sighted to place overriding emphasis on altruism as the guiding principle. He suggested that solidaristic donation—motivated by feelings of social or group-focused solidarity—seems to encompass altruism as well as other acceptable motivations [28]. The present study supported the argument that a broader definition of acceptable motivations is appropriate and would perhaps open the door to a larger pool of donors.

There is an apparent assumption held by many staff members that SKDrs choose to donate purely out of love and loyalty to their loved one or family member. Conversely, the motives of UKDrs are regarded with suspicion and interrogated more intensely by some members of the medical team. Whilst we acknowledge that SKDrs may derive more benefit than UKDrs due to their personal connection with the recipient, we question whether the more critical approach towards UKDrs motivations by the medical team is justified or logical. Many staff members, whilst acknowledging the importance of the rigorous assessment of UKD, noted that the same standards were not always applied to SKDrs and questioned whether they should be, due to potential issues such as guilt, family obligation, manipulation or reciprocity. Some authors have even suggested restraint of the LKD programme because of the possible social and familial tensions it may provoke [29]. To date there has been very little research on the complex family dynamics of LKD but what little literature does exist demonstrates that feelings of obligation, psychological distress and social-familial alienation following donations are very real [30, 31]. There is an argument to be made that assessment of SKDrs should be brought up to the same rigorous standards to that of UKDrs.

The traditional mindset, documented in previous literature [32, 33], that UKDrs are driven by a form of psychopathology, was also suggested by the current study. Our study demonstrated that there is still a lot of negativity towards UKD, and thus the need to educate individuals towards a more open-minded mentality towards all living kidney donors. Whilst there is not a strong body of evidence affirming the psychological wellbeing of UKDrs, neither is there evidence of an underlying psychopathology. Previous research demonstrates that UKDrs have positive outcomes [34] and equivalent psychological outcomes to SKDrs [9, 35]. Motives are honourable, however the evidence to date for the personal benefit of UKD is mixed [36], and studies reporting benefits are mainly retrospective [37]. The BOUnD study will hopefully help to fill this gap in the literature [8]. We feel strongly that further training amongst staff is necessary to develop a consistent and affirmative approach to UKDrs at all centres. A concerted effort to increase healthcare professionals’ awareness of the value of UKDrs, and to address their concerns, would greatly strengthen the overall programme.

A previous study investigating the experiences of completed, medically and self-withdrawn donors [38], found that some potential UKDrs who self-withdrew from the programme reported that they did so because of their impression that some healthcare staff were against them subjecting themselves to surgery. However, in this study, staff members did not perceive that their personal opinions were a factor in self-withdrawal. The clinical implication of this disconnect would be to ensure that the staff’s private opinions do not affect their treatment of donors or influence the way they communicate with them. It is important for staff members to present a consistent and unbiased position even if they have personal reservations about UKD. Should professionals strongly object to UKD, it may be advisable to consider whether professionals should be allowed to conscientiously object to being involved. Such a system would allow healthcare professionals to choose to opt out from the practice if it goes against their personal beliefs and values.

Many staff members expressed the view that donor expectations needed to be managed, specifically when it comes to the issue of anonymity. There is however a larger discussion amongst UKD programmes globally around whether or not the condition of anonymity should be revisited [10, 26, 39, 40]. In one of the few qualitative studies of physicians’ attitudes towards UKD, Fortin et al. (2008) found considerable opposition to lifting the strict requirement for anonymity [41]. This is in line with the current study, which found that some staff acknowledged that some UKDrs struggled with the requirement for anonymity, principally due to a strong psychological need for connection with the recipient. This correlates with a paper by Pronk et al which identified that some UKDrs remained troubled by and curious about the lack of contact with their recipient many years after their donation [42]. Future studies need to probe this issue both from the perspective of the recipient as well as the donor to determine if there is a mutual reciprocal benefit that challenges the current rules around anonymity. It should be noted that for a donor, the ability to know the outcome of the donation does not contradict the principle of altruism. Rather, knowledge of the outcome may relate to the need for closure.

### Strengths and Limitations

The strengths of this study lie in the number of interviews which allowed for data saturation. It is acknowledged that the data were collected 7 years ago from only six centres, and that transplant professionals’ perspectives could have evolved since. However, a significant shift in either positive or negative views or opinions does not appear apparent within the academic or clinical environment.

Many participants, particularly those working in low volume transplant centres, acknowledged that they had only minimal clinical experience working with UKDrs. Consequently, these interviews were much shorter than those conducted in higher volume centres, however the overall impressions were similar. Additionally, opportunistic sampling is a limitation which should be addressed in future research. However, the sample in our study was still representative of the transplant community. Finally, we were not able to adjust for interviewees’ exposure. Despite these limitations, this is the first qualitative study to assess the approach of transplant professionals towards UKDrs in depth and as such offers valuable insights.

This paper is applicable to other areas of transplantation, and indeed the wider healthcare setting, by acknowledging the relationship between professionals’ views and the impact of their subconscious communication to patients. Participants in this study were explicitly asked whether they felt they unduly influenced UKDrs during their interactions with them and reported that they did not. However, a study conducted simultaneously within a group of donors and withdrawn donor candidates who would have been cared for by some of these same individuals reported differently. Healthcare professionals ought to be mindful of how their views may negatively influence patients in the clinical environment as they may not be fully aware of their impact.

### Conclusion

This study provides valuable insight into the practice of UKD and has identified key areas which need addressing. There needs to be clarity on the age limit policy for each transplant centre, a discussion around the necessity of formal psychological assessment for all living kidney donors, and a new approach to managing UKDrs’ expectations, particularly around anonymity. Specific suggestions are to enhance training and improve consistency between all members of the multidisciplinary teams across all UK transplant centres. Implementing these findings will strengthen the practices towards LKD, improve the donation experience for everyone involved, and result in an increased acceptance of unspecified donation as a key element in the kidney transplant programme.

## Data Availability

The raw data supporting the conclusion of this article will be made available by the authors, without undue reservation.

## Abbreviations

BOUnD, Barriers and Outcomes in Unspecified Donation; HTA, human tissue authority; LKD, living kidney donation; NHS, National Health Service; SKD, specified kidney donation; SKDrs, specified kidney donors; UKD, unspecified kidney donation; UKDrs, unspecified kidney donors; UKLKSS, UK Living Kidney Sharing Scheme; UK, United Kingdom.
